# Estimated 24-h urinary sodium excretion and risk of end-stage kidney disease

**DOI:** 10.1016/j.isci.2023.106728

**Published:** 2023-04-23

**Authors:** Ying Shan, Yong Bai, Jingwen Zhang, Yueqi Lu, Sike Yu, Congying Song, Juehan Liu, Min Jian, Junjie Xu, Changhai Ding, Zuying Xiong, Xiaoyan Huang

**Affiliations:** 1 Clinical Research Academy, Peking University Shenzhen Hospital, Peking University, Shenzhen 518036, China; 2 BGI-Shenzhen, Shenzhen 518083, China; 3 Renal Division, Peking University Shenzhen Hospital, Peking University, Shenzhen 518036, China; 4 Clinical Research Centre of Zhujiang Hospital, Southern Medical University, Guangzhou 510280, China

**Keywords:** Health sciences, Nephrology, Human Physiology

## Abstract

The association between sodium intake and long-term kidney disease endpoints is debated and yet to be proven. We aimed to investigate the associations of estimated 24-h urinary sodium excretion, reflecting daily sodium intake, with the incidence of end-stage kidney disease (ESKD). In this prospective cohort study including 444,375 UK Biobank participant, 865 (0.2%) ESKD events occurred after median follow-up of 12.7 years. For every 1 g increment in estimated 24-h urinary sodium excretion, multivariable-adjusted hazard ratio for incident ESKD was 1.09 (95% confidence interval 0.94–1.26). Nonlinear associations were not detected with restricted cubic splines. The null findings were confirmed by a series of sensitivity analyses, which attenuated potential bias from measurement errors of the exposure, regression dilution, reverse causality, and competing risks. In conclusion, there is insufficient evidence that estimated 24-h urinary sodium excretion is associated with the incidence of ESKD.

## Highlights

• Evidence suggests that sodium reduction lowers blood pressure and albuminuria
• The link between sodium intake and end-stage kidney disease (ESKD) is unproven
• We observed that urinary sodium excretion was not associated with incident ESKD
• Low sodium intake may not prevent ESKD in a relatively healthy population

## Graphical abstract

**Figure:**
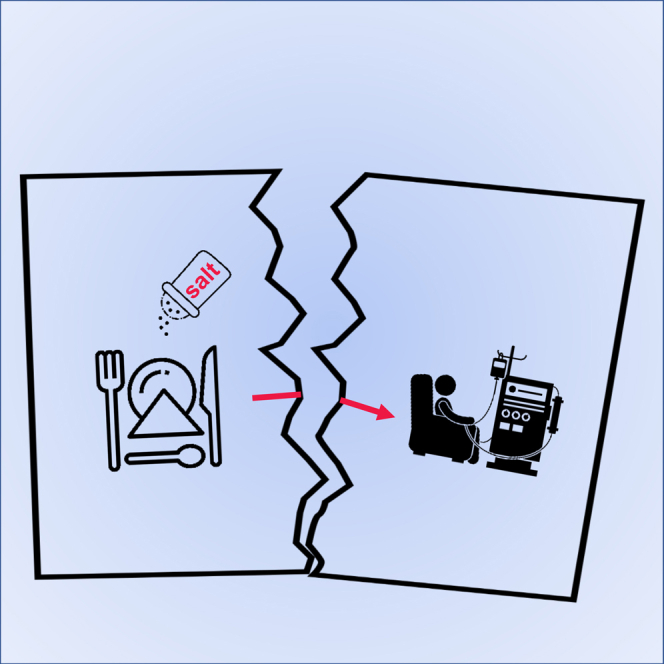

## Introduction

Current guidelines almost uniformly propose low sodium intake in the entire population.^1^^,^^2^^,^^3^ Historically, this recommendation relied on convincing evidence that high sodium intake causally increases blood pressure, as well as on the inference that interventions to reduce blood pressure would subsequently decrease target organ damage.^1^ Whether the effects of manipulating sodium intake could successfully translate to the expected benefits remained inconclusive for a long time.^4^^,^^5^ Until recently, high-quality evidence that ascertains the relationship of sodium intake with cardiovascular events and death was revealed.^6^^,^^7^ However, somewhat unexpectedly, dietary sodium restriction did not reduce the composite of cardiovascular events in patients with heart failure.^8^ Altogether, evidence to date generally supports the benefit of lowering sodium intake on human health, but more efforts should be devoted to a broader population or specific outcomes.^9^

Uncontrolled high blood pressure has long been endorsed as an important risk factor for the initiation and progression of kidney diseases. In addition, albuminuria serves as another key determinant of progressive kidney function loss.^10^ Akin to its impact on blood pressure, sodium reduction ameliorates urinary excretion of albumin in people with chronic kidney disease (CKD).^11^^,^^12^ Through causal pathways involving blood pressure and/or albuminuria, excess sodium consumption is thus presumed to drive the long-term progression of kidney impairment. However, robust evidence suggesting low sodium intake delays the progression of kidney function decline is lacking.^9^^,^^11^^,^^13^^,^^14^ Previous results of this kind have been mainly obtained in patients with overt CKD. Thus, the association between sodium intake and incident end-stage kidney disease (ESKD) is still debated, particularly in those at low- or moderate-risks. Data from a large-scale population-based cohort would be of great value to elucidate the controversy about the hypothesized but unproven benefit of sodium reduction on kidney endpoints.

In this study, we investigated the linear and nonlinear associations of estimated 24-h urinary sodium excretion, reflecting daily sodium intake, with the incidence of ESKD in 444,375 community-dwelling UK Biobank participants. What is more, many cohort studies typically utilizing a single measurement of sodium intake failed to capture the combined effects of its random errors and long-term fluctuations within persons. Such a phenomenon, i.e., regression dilution bias,^15^ inevitably introduces downward bias of the estimated association between sodium and diseases. Data on a repeated measurement of urinary biomarkers over years were also available in a subsample of the UK Biobank participants, enabling us to address the issue of regression dilution bias. Several sensitivity analyses were also undertaken to confirm the main finding.

## Results

### Baseline characteristics

**Figure 1:**
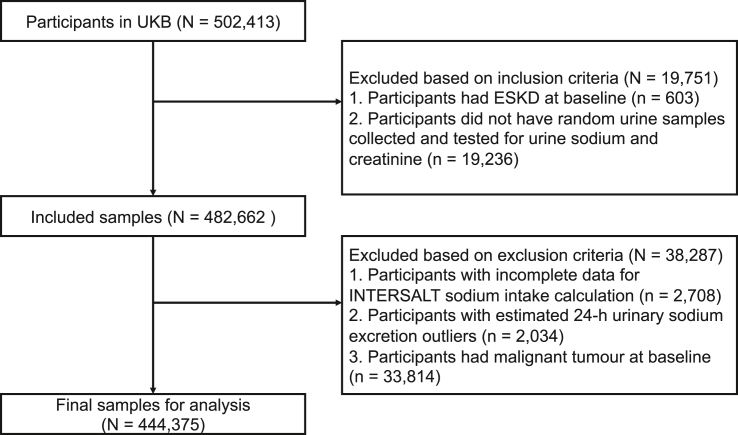
Flow chart of inclusion and exclusion ESKD, end-stage kidney disease; UKB, UK Biobank.

Of the 502,413 UK Biobank participants, 58,038 were excluded based on the inclusion and exclusion criteria, leaving 444,375 participants eligible for analyses in the study (Figure 1). The mean (SD) estimated 24-h urinary sodium excretion was 3.3 (0.8) g calculated by INTERSALT equations and 4.1 (1.2) g calculated by Kawasaki equations (Figure S1). Only 2.1% and 2.7% of the participants met the World Health Organization recommendation that sodium intake should be less than 2 g/d, estimated by the INTERSALT and Kawasaki equations, respectively. After a mean (SD) of 4.3 (0.9) years, repeated measurements of the urinary biomarkers were available in a subsample of 17,205 UK Biobank participants who met the inclusion criteria.

Table 1 shows the baseline characteristics of the study participants collectively, as well as grouped by quartiles of estimated 24-h urinary sodium excretion before the multiple imputations. Among the 444,375 participants, the mean age was 56.2 years, and 54.1% were females. The overall mean estimated 24-h urinary sodium excretion was higher in males than in females (4.0 g versus 2.8 g). In summary, people in the lowest estimated 24-h urinary sodium excretion quartile (Q1) were more likely to be older, white, have a lower Townsend deprivation index, and have a higher education level than those in the highest quartile (Q4). In addition, people in Q1 had a lower likelihood of smoking, alcohol consuming, being obese, and had lower estimated 24-h urinary potassium excretion, blood pressure, and UACR levels. They had a lower prevalence of comorbidities, including hypertension, diabetes, CHD, CHF, and stroke. They are also less likely to be on certain medications, such as diuretics, ACEIs, and ARBs.

**Table 1:** Baseline characteristics of participants

Variable	Missing value, n	Whole population	Estimated 24-h urinary sodium excretion (g)			
			Q1 [0.81, 2.68]	Q2 (2.68, 3.2]	Q3 (3.2, 3.92]	Q4 (3.92, 5.84]
n		444375	111094	111094	111093	111094
Age, year	0	56.24 ± 8.10	59.08 ± 7.43	54.31 ± 7.76	54.96 ± 8.19	56.61 ± 8.15
Females	0	240241 (54.06)	106313 (95.70)	96367 (86.74)	33231 (29.91)	4330 (3.90)
Ethnicity	2083					
White		418006 (94.51)	106880 (96.56)	104262 (94.22)	103244 (93.44)	103620 (93.82)
Asian		10282 (2.32)	1440 (1.30)	2668 (2.41)	3146 (2.85)	3028 (2.74)
Black		7256 (1.64)	1072 (0.97)	1820 (1.64)	2292 (2.07)	2072 (1.88)
Others		6748 (1.53)	1301 (1.18)	1906 (1.72)	1812 (1.64)	1729 (1.57)
Townsend deprivation index	544	−2.14 [-3.65 0.53]	−2.37 [-3.75 0.01]	−2.12 [-3.63 0.50]	−2.07 [-3.62 0.73]	−1.96 [-3.55 0.87]
Education^a^	6473					
Level 1		73716 (16.83)	19770 (18.03)	16178 (14.76)	15775 (14.40)	21993 (20.15)
Level 2		118100 (26.97)	29166 (26.61)	31592 (28.82)	28507 (26.03)	28835 (26.42)
Level 3		101337 (23.14)	23946 (21.84)	24704 (22.54)	25419 (23.21)	27268 (24.98)
Level 4		144749 (33.06)	36742 (33.52)	37139 (33.88)	39816 (36.36)	31052 (28.45)
IPAQ activity group	85524					
Low		66898 (18.64)	13703 (15.69)	16601 (18.85)	17593 (19.18)	19001 (20.71)
Moderate		146321 (40.77)	37661 (43.12)	37500 (42.59)	36456 (39.75)	34704 (37.82)
High		145632 (40.58)	35967 (41.18)	33952 (38.56)	37662 (41.07)	38051 (41.47)
Smoking status	2160					
Never		243682 (55.10)	65924 (59.60)	65319 (59.06)	60765 (54.96)	51674 (46.79)
Previous		151640 (34.29)	35299 (31.91)	34260 (30.98)	36604 (33.11)	45477 (41.18)
Current		46893 (10.60)	9394 (8.49)	11020 (9.96)	13200 (11.94)	13279 (12.02)
Alcohol consumption	1059					
Never		19458 (4.39)	5685 (5.13)	5593 (5.05)	4668 (4.21)	3512 (3.17)
Occasional		15453 (3.49)	3890 (3.51)	3648 (3.29)	3828 (3.45)	4087 (3.69)
Frequent		408405 (92.13)	101310 (91.36)	101612 (91.66)	102302 (92.33)	103181 (93.14)
Estimated 24-h urinary potassium excretion, g	0	2.75 ± 0.59	2.60 ± 0.57	2.67 ± 0.56	2.81 ± 0.60	2.93 ± 0.58
Urinary sodium-to-potassium ratio	0	1.25 ± 0.36	0.95 ± 0.22	1.14 ± 0.26	1.32 ± 0.29	1.57 ± 0.34
Body mass index, kg/m^2^	0	27.37 ± 4.70	24.63 ± 3.56	27.07 ± 4.07	27.78 ± 4.87	30.02 ± 4.54
Waist circumference, cm	76	90.14 ± 13.25	80.04 ± 10.12	85.92 ± 10.86	93.14 ± 10.32	101.45 ± 10.92
Hypertension history	1521	104143 (23.52)	21940 (19.81)	23083 (20.84)	24908 (22.51)	34212 (30.92)
Diabetes history	1870	22349 (5.05)	2834 (2.56)	3899 (3.52)	5709 (5.16)	9907 (8.97)
Coronary heart disease history	1521	19689 (4.45)	3040 (2.74)	2834 (2.56)	5340 (4.82)	8475 (7.66)
Congestive heart failure history	0	1787 (0.40)	222 (0.20)	210 (0.19)	490 (0.44)	865 (0.78)
Stroke history	1521	5191 (1.17)	1220 (1.10)	1000 (0.90)	1291 (1.17)	1680 (1.52)
Diuretics	0	31799 (7.16)	6469 (5.82)	7696 (6.93)	6909 (6.22)	10725 (9.65)
ACEIs or ARBs	0	69188 (15.57)	12372 (11.14)	12936 (11.64)	16699 (15.03)	27181 (24.47)
Glucocorticoids	0	3549 (0.80)	937 (0.84)	846 (0.76)	887 (0.80)	879 (0.79)
eGFR, mL/min/1.73m^2^	25324	88.80 ± 15.95	87.51 ± 15.31	90.75 ± 15.85	89.88 ± 16.01	87.08 ± 16.30
eGFR<60 mL/min/1.73m^2^	25324	17480 (4.17)	4741 (4.53)	3773 (3.61)	3863 (3.69)	5103 (4.86)
UACR	14055					
<30 mg/g		410822 (95.47)	100675 (95.88)	101102 (96.01)	105034 (95.85)	104011 (94.18)
30-300 mg/g		17851 (4.15)	4052 (3.86)	3903 (3.71)	4129 (3.77)	5767 (5.22)
≥300 mg/g		1647 (0.38)	277 (0.26)	301 (0.29)	414 (0.38)	655 (0.59)

Mean ± standard deviation, median [interquartile range], and frequency (percentage) are shown for symmetrical continuous variables, skewed continuous variables, and categorical variables, respectively.

ACEIs, angiotensin-converting enzyme inhibitors; ARBs, angiotensin II receptor blockers; eGFR, estimated glomerular filtration rate; IPAQ, the International Physical Activity Questionnaires; UACR, urine albumin-to-creatinine ratio.

a Education: level 1, “none of the above”; level 2, “O levels/GCSEs or equivalent” or “CSEs or equivalent”; level 3, “A levels/AS levels or equivalent” or “NVQ or HND or HNC or equivalent” or “Other professional qualifications”; level 4, “College or University degree”.

### Estimated 24-h urinary sodium excretion and incident ESKD

The median follow-up period was 12.7 (interquartile range 12.0–13.4) years. During follow-up, 1,130 (0.3%) participants lost to follow-up, 865 (0.2%) participants progressed to ESKD, and 29,252 (6.6%) participants died. The incidence rate was 15.6 per 100,000 person-years for ESKD.

**Figure 2:**
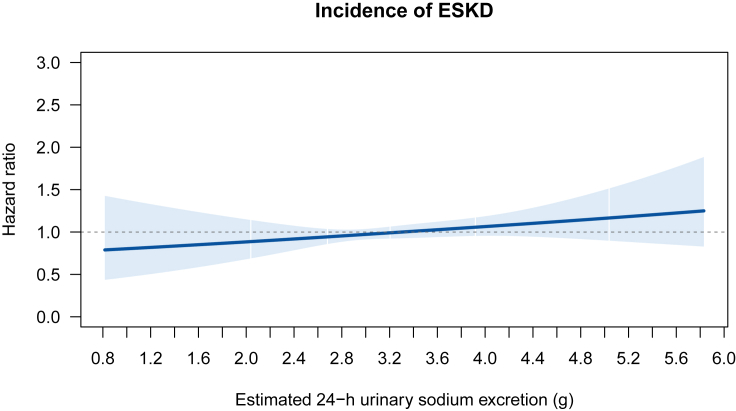
Restricted cubic splines for the incidence of end-stage kidney disease The analyses were adjusted for age, sex, ethnicity, education, Townsend deprivation index, smoking, alcohol consumption, physical activity, estimated 24-h urinary potassium excretion, waist circumference, hypertension, diabetes, coronary heart disease, congestive heart failure, stroke, diuretics, angiotensin-converting enzyme inhibitors, angiotensin II receptor blockers, and estimated glomerular filtration rate. Each point of the curve is the pointwise average hazard ratio. Shaded areas represent 95% confidence intervals. White vertical lines through confidence shade represent 2.5^th^ percentile, first quartile, median, third quartile, and 97.5^th^ percentile of estimated 24-h urinary sodium excretion. ESKD, end-stage kidney disease.

The main association results were pooled from the estimates in 5 complete datasets after multiple imputations following Rubin’s rule. No multicollinearity was found in all the models presented. The regression dilution ratio (RDR) was 0.84. As shown in Table 2, in crude models, the continuous form and the discrete form of estimated 24-h urinary sodium excretion appeared to be positively associated with incident ESKD. Next, we examined the association between estimated 24-h urinary sodium excretion and incident ESKD, adjusted for potential confounders in Model 1 and 2. Model 1 reached similar conclusions to the crude analysis but with gradually attenuated estimates. The fully adjusted model, model 2, indicated that associations between all forms of estimated 24-h urinary sodium excretion and incident ESKD were statistically nonsignificant. In Model 2, the RDR-adjusted hazard ratios (HRs) (95% confidence intervals [CIs]) were 1.09 (0.94, 1.26) and 1.61 (0.90, 2.89) for continuous and binary forms of estimated 24-h urinary sodium excretion, respectively. The RDR-adjusted HRs (95% CI) of Q4 was 1.21 (0.85, 1.71) as compared with Q1. The restricted cubic spline shown in Figure 2 was consistent with these results. The HRs for incident ESKD were not significantly different from one, with the confidence intervals overlapping the null across the whole spectrum of estimated 24-h urinary sodium excretion. Also, the restricted cubic spline analysis showed that it did not appear to be any nonlinear relationship with P for nonlinearity of 0.93.

**Table 2:** Incidence rates and hazard ratios for end-stage kidney disease

Estimated 24-h urinary sodium excretion	Incidence rate, per 100,000 person-years	Crude			Model 1			Model 2		
		HRs (95% CIs)	RDR-adjusted HRs (95% CIs)	p	HRs (95% CIs)	RDR-adjusted HRs (95% CIs)	p	HRs (95% CIs)	RDR-adjusted HRs (95% CIs)	p
Continuous, /1g increment	15.6	1.56 (1.44, 1.68)	1.69 (1.54, 1.86)	<0.001	1.26 (1.13, 1.41)	1.32 (1.15, 1.51)	<0.001	1.07 (0.95, 1.21)	1.09 (0.94, 1.26)	0.255
Binary										
Below 2g	14.6	Reference	Reference		Reference	Reference		Reference	Reference	
Above 2g	15.6	1.07 (0.66, 1.72)	1.08 (0.61, 1.91)	0.793	1.14 (0.70, 1.85)	1.16 (0.65, 2.08)	0.607	1.49 (0.91, 2.44)	1.61 (0.90, 2.89)	0.111
Multicategory										
Quartile 1	10.3	Reference	Reference		Reference	Reference		Reference	Reference	
Quartile 2	10.6	1.03 (0.82, 1.29)	1.03 (0.79, 1.36)	0.821	1.16 (0.92, 1.47)	1.20 (0.90, 1.58)	0.209	0.99 (0.78, 1.26)	0.99 (0.75, 1.31)	0.955
Quartile 3	17.2	1.67 (1.36, 2.05)	1.84 (1.44, 2.35)	<0.001	1.36 (1.05, 1.77)	1.44 (1.06, 1.97)	0.021	1.11 (0.86, 1.44)	1.14 (0.84, 1.54)	0.406
Quartile 4	24.5	2.38 (1.96, 2.89)	2.80 (2.22, 3.53)	<0.001	1.57 (1.19, 2.09)	1.71 (1.23, 2.39)	0.002	1.17 (0.87, 1.57)	1.21 (0.85, 1.71)	0.290
P for linear trend		<0.001			0.002			0.217		
P for quadratic trend		0.023			0.965			0.669		

Model 1 was adjusted for age, sex, ethnicity, Townsend deprivation index, education, smoking status, alcohol consumption, physical activity, and estimated 24-h urinary potassium excretion.

Model 2 was adjusted for age, sex, ethnicity, Townsend deprivation index, education, smoking status, alcohol consumption, physical activity, estimated 24-h urinary potassium excretion, waist circumference, hypertension history, diabetes history, coronary heart disease history, stroke history, diuretics, angiotensin-converting enzyme inhibitors or angiotensin II receptor blockers, and estimated glomerular filtration rate. The regression dilution ratio was 0.84.

CIs, confidence intervals; HRs, hazard ratios; RDR, regression dilution ratio.

### Sensitivity and subgroup analyses

The result that estimated 24-h urinary sodium excretion was not significantly associated with ESKD was supported by sensitivity and subgroup analyses. As shown in Tables S1–S8, the RDR-adjusted HRs (95% CIs) were 0.94 (0.72,1.22), 1.11 (0.91,1.36), 1.28 (0.74,2.24), 0.83 (0.55,1.26), 1.07 (0.94, 1.23), 1.10 (0.92, 1.31), 1.09 (0.95, 1.27) and 1.13 (0.97,1.33), respectively, for continuous form of estimated 24-h urinary sodium excretion under eight conditions. Similar null results were detected when we treated the exposures in the binary or multicategorical forms. Table S9 shows subgroup analysis stratified by sex. The RDR-adjusted HRs (95% CIs) were 0.77 (0.50,1.19) and 1.14 (0.86,1.52) for women and men, respectively. No statistical significance between sex-specific association (p = 0.07) was observed using a 2-sample z-test.

## Discussion

In this large prospective population-based cohort study, we observed no linear or nonlinear associations between estimated 24-h urinary sodium excretion and future ESKD in 444,375 UK Biobank participants followed for over 12 years. This finding was confirmed by the correction for regression dilution bias and several sensitivity analyses, which attenuated potential bias from measurement errors and/or long-term intra-individual variability of the exposure, reverse causality, and competing risks. The null results add to the literature that low sodium consumption may not suffice to translate to clinically significant reductions in the risk of ESKD.

Previous cohort study showed that for the population at high risk of future kidney-related events, high urinary sodium excretion was linked with CKD progression and ESKD incidence. He et al.^16^ reported a strong association between >4.4 g/d urinary sodium excretion (highest quartile) and CKD progression and all-cause mortality in patients with established CKD, in the Chronic Renal Insufficiency Cohort Study. In CKD and general populations, randomized trials have shown that sodium restriction (<2.3 g/d) reduces blood pressure and albuminuria in CKD and general populations,^17^^,^^18^^,^^19^^,^^20^ without a beneficial effect on kidney function being reported.^18^^,^^19^^,^^20^^,^^21^^,^^22^^,^^23^ Nevertheless, these interventional studies are typically limited by sample size and short duration. A recent Cochrane systematic review evaluated 21 randomized controlled trials published through October 2020, involving a total of 1,197 adults with CKD.^12^ The average study duration was only 7 (range: 1–36) weeks. These studies consequently had to focus on surrogate markers rather than hard endpoints. As a result, intervention studies to date have provided little evidence directly linking sodium restriction with long-term kidney outcomes, albeit the findings of lowering blood pressure and albuminuria in the short term are of high certainty.

The results of kidney disease progression therefore have to be largely derived from observational studies. Previous publications, however, show conflicting results investigating the association between sodium intake and subsequent onset or progression of CKD. Several studies observed that high sodium intake was associated with future ESKD, halving of eGFR from baseline, or risk of death,^16^^,^^24^^,^^25^^,^^26^^,^^27^^,^^28^ but the findings could not be replicated by others,^29^^,^^30^^,^^31^ including a two-sample Mendelian randomization analysis.^32^ For this reason, limited and inconsistent evidence supports an association between high sodium intake and kidney outcomes.^11^^,^^33^ The large discrepancy in findings in the CKD population is attributable, at least to some extent, to reverse causality^34^ and measurement errors.^35^ Reverse causality, introduced by the fact that overt CKD patients may either eat less or adhere to the recommendation of low sodium intake, tends to lead to underestimation of the sodium-kidney outcome relation.^34^ Furthermore, the ability to excrete sodium decreases with progressive decline in kidney function, compromising the reliability of urinary biomarker measurement, which is often utilized as a metric of sodium intake in epidemiological research.^35^Data in the non-CKD population are less prone to these biases. Even though somewhat mixed,^36^ most results propose that high sodium intake is associated with developing CKD in high-risk individuals,^37^^,^^38^^,^^39^ as well as with longitudinal changes in eGFR.^40^^,^^41^ Nevertheless, the relatively small sample size may have prevented researchers from further determining the association between sodium intake and incident ESKD in the previous literature.^42^^,^^43^

**Figure 3:**
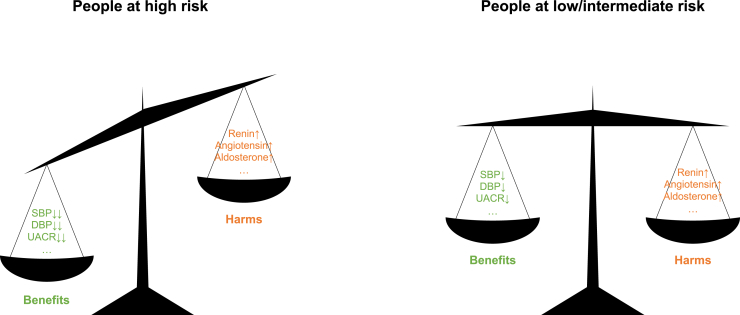
Proposed interpretation of the net effects of low sodium intake in different people Low sodium intake leads to a reduction in levels of blood pressure and albuminuria but comes at the cost of certain adverse risks, such as higher plasma renin and aldosterone concentrations. In people at high risk, the benefits of low sodium intake exceed the harms; whereas the harms may counteract the benefits in people at low- or intermediate risk (e.g., without hypertension or albuminuria). DBP, diastolic blood pressure; SBP, systolic blood pressure; UACR, urine albumin-to-creatinine ratio.

We found that sodium intake is not a risk factor, or is solely a minor risk factor compared with other established risk factors for incident ESKD. The reasons have not been fully elucidated. One possible interpretation is, as depicted in Figure 3, that sodium restriction leads to a reduction in levels of blood pressure and albuminuria but comes at the cost of higher plasma renin and aldosterone concentrations. Evidence of such sideeffects is consistent, indicating a compensatory response to a decrease in effective circulating volume caused by sodium restriction, especially in persons with normal blood pressure.^44^ Both renin^45^^,^^46^ and aldosterone^47^ can directly cause kidney damage, in turn counteracting the potential benefits via favorable effects of sodium restriction on blood pressure and albuminuria. Specifically, our study cohort mostly consisted of relatively healthy participants, with approximately 90% and 95% of them being normotensive and normoalbuminuric, respectively. The net effect of sodium intake tended to be neutral after balancing its beneficial and detrimental effects, as observed in such a community-dwelling population.

In conclusion, this population-based cohort study suggests that estimated 24-h urinary sodium excretion is not linearly or nonlinearly associated with the incidence of ESKD. Our finding nonetheless does not support the hypothesized notion that low sodium intake prevents ESKD, at least in individuals without hypertension and albuminuria.

### Limitations of the study

The “gold standard” method for estimating sodium intake is repeated 24-h urine collection. However, because of the difficulty in such a very large population cohort with multiple study centers and a central biobank, the UK Biobank collected random urinary spot samples. We had employed both the INTERSALT and Kawasaki equations, which led to cross-validation somewhat because the bias of both approaches might not be identical.^48^ We could not completely exclude the risk of committing a type II error, particular in the context that another UK Biobank publication did not observe an association of estimated urinary sodium excretion with all-cause mortality and fatal or nonfatal cardiovascular disease events either.^49^ Despite the significant associations in model 1, they diminished when anthropometric parameter was additionally included as a covariate in our final models. This might indicate intrinsic pitfalls of the equations we employed.^50^ As such, potential methodological limitations need to be considered in interpreting our findings. Nevertheless, the exposure appeared linearly associated with blood pressure and UACR, i.e., positive controls,^17^^,^^18^^,^^19^^,^^20^ in the current analysis. Furthermore, the use of urinary sodium-to-potassium ratio, exempt from the aforementioned mathematical flaws and also valuable to approximate sodium intake in large surveys,^51^ generated results in concert with the main finding. Efforts of this kind might have reduced the probability that exposure misclassification biased our results to the null.

Apart from the lack of measured 24-h urinary sodium excretion, other limitations of our study should also be acknowledged. First, given the observational nature of the data, we described associations rather than inferring causality because reverse causality and residual confounding were impossible to completely avoid. Second, participants recruited to the UK Biobank were volunteers and, thus, may not be representative of the general population. In addition, the vast majority of participants were of European white descent despite the inclusion of other ethnicities. Hence, our results should be interpreted cautiously and await confirmation in other ethnic groups with significantly different diets or prevalence of and predispositions to kidney diseases. Lastly, repeat eGFR measures over the follow-up period were not available for most UK Biobank participants, preventing us from further investigating the association of sodium intake with trajectories of kidney function decline.

## STAR★Methods

### Key resources table

**Table:** 

REAGENT or RESOURCE	SOURCE	IDENTIFIER
**Depositeddata**		

UK Biobank	UK Biobank	https://www.ukbiobank.ac.uk/

**Software and algorithms**		

R 4.1.3	The R Foundation for Statistical Computing	https://www.r-project.org/
cmprsk (R)	Open source	https://cran.r-project.org/web/packages/cmprsk/index.html
dplyr (R)	Open source	https://cran.r-project.org/web/packages/dplyr/index.html
Hmisc (R)	Open source	https://cran.r-project.org/web/packages/Hmisc/index.html
mice (R)	Open source	https://cran.r-project.org/web/packages/mice/index.html
rms (R)	Open source	https://cran.r-project.org/web/packages/rms/index.html
survival (R)	Open source	https://cran.r-project.org/web/packages/survival/index.html
survminer (R)	Open source	https://cran.r-project.org/web/packages/survminer/index.html
Codes in this paper	This paper	https://zenodo.org/badge/latestdoi/624769968

### Resource availability

#### Lead contact

Further information and requests for resources and reagents should be directed to and will be fulfilled by the lead contact, Xiaoyan Huang (huangxiaoyan@pku.org.cn).

#### Materials availability

This study did not generate new unique reagents.

### Experimental model and subject details

UK Biobank is a large-scale biomedical database and research resource. The UK Biobank received ethical approval from the North West Multi-Center Research Ethics Committee (REC reference: 11/NW/03820). The study was conducted in accord with the principles of the Declaration of Helsinki. Our study employed a cohort design using UK Biobankdata and did not involve a clinical trial; therefore, no clinical registry number or associated links are available for reference.

The UK Biobank comprises 502,413 volunteers aged 40 to 69 years (around 54% of female and 94% of self-reported European ancestry) recruited through the United Kingdom National Health Service registers between April 2007 and December 2010. Participants attended 1 of 22 dedicated assessment centers in England, Scotland, and Wales, as described elsewhere.^52^ The participants completed a computer-based questionnaire on demographics, life-course exposures, self-reported health behavior, medical history, and treatments. Additionally, participants underwent a standardized portfolio of clinical examinations and assays of biological samples. Biological samples were also collected in a subsample of participants during 2012 and 2013.

### Method details

#### Study design

The present study is a prospective cohort study based on UK Biobank participants. The study hypothesis arose before inspection of the data. The study hypothesis arose before inspection of the data and a research plan had been submitted to the UK Biobank (application number 73684). However, no protocol for the present analysis was published. Variables used specifically in this work are summarized in Table S10. The process of inclusion and exclusion is depicted in Figure 1. This study included participants with available data on urine sodium and creatinine and without prevalent end-stage kidney disease (ESKD) at baseline according to a prespecified algorithm (https://biobank.ndph.ox.ac.uk/showcase/ukb/docs/alg_outcome_esrd.pdf, last accessed April 4^th^, 2022). The exclusion criteria were incomplete data (age, sex, height, weight, etc.) for estimating 24-h urinary sodium excretion, outliers of estimated 24-h urinary sodium excretion (beyond mean ± 5SD), and prevalent malignant tumor at baseline.

#### Exposure estimates

A spot midstream urine sample was obtained at the end of a 2-h visit and refrigerated between 2°C and 8°C. All urinary biomarkers were measured on a single Beckman Coulter AU5400 clinical chemistry analyzer using the manufacturer’s reagents and calibrators, except for urinary albumin, for which reagents and calibrators were sourced from Randox Bioscience. The Beckman Coulter AU5400 analyzer used a potentiometric measurement for the determination of sodium and potassium concentrations and a photometric measurement for the determination of creatinine and albumin concentrations. The analysis method for urinary sodium and potassium involved a sample predilution step, while for urinary albumin and creatinine assays, it allowed samples with results exceeding the upper analytical limit of the assay to be diluted and reanalyzed. Internal quality control was performed for all these urinary biomarkers (https://biobank.ndph.ox.ac.uk/showcase/ukb/docs/urine_assay.pdf, last accessed April 4^th^, 2022).

The primary exposure was 24-h urinary sodium excretion (g) estimated from the spot urinary biomarker concentrations based on the sex-specific International Cooperative Study on Salt, Other Factors, and Blood Pressure (INTERSALT) equations with a Western Europe intercept.^53^ The INTERSALT equations, adopted in another study based on the UK Biobankdata,^54^ are the least biased compared with other predictive equations, including the Kawasaki equations. Unlike the Kawasaki equations, the INTERSALT equations have been developed using nonfasting spot urine samples as obtained in the UK Biobank. We also used the Kawasaki-based estimated 24-h urinary sodium excretion and the urinary sodium-to-potassium ratio as secondary exposures in sensitivity analyses.^55^^,^^56^

#### Ascertainment of ESKD

Both retrospective and prospective data linked to electronic health records, including hospital episode statistics data on diagnoses (the International Classification of Diseases codes) and operations (the Office of Population Censuses and Surveys Classification of Interventions and Procedures codes) and cause of death (the International Classification of Diseases codes) data through the Office for National Statistics, are available in the UK Biobank. The follow-up period of the present study started on the date of first assessment and ended on the date of death, first date of ESKD diagnosis, the end of follow-up, or November 12^th^, 2021, whichever occurred first. The outcome of interest was incident ESKD, as per an algorithm (https://biobank.ndph.ox.ac.uk/showcase/ukb/docs/alg_outcome_esrd.pdf, last accessed April 4^th^, 2022) prespecified by the UK Biobank Outcome Adjudication Group. In brief, an algorithm first identifying UK Biobank participants who had received kidney replacement therapy (dialysis or kidney transplantation) was devised, and the subset with other relevant diagnostic or procedural codes specific to ESKD (indicators of CKD stage 5) was then selected. The principles used in this algorithm have previously been used to successfully identify people with treated ESKD in a UK cohort.^57^

#### Covariates

Covariates were 1). Demographic factors, including age, sex, ethnicity (categorized into white, Asian, black, and others), education (categorized into level 1, level 2, level 3, and level 4), and Townsend deprivation index; 2). Lifestyle factors, including smoking, alcohol consumption, physical activity (categorized into low, moderate, and high evaluated with the International Physical Activity Questionnaires), and estimated 24-h urinary potassium excretion (representing daily potassium intake; calculated by the Kawasaki equation)^55^; 3). Anthropometric and clinical measurements, including body mass index, waist circumference, systolic blood pressure, and diastolic blood pressure; 4). Comorbid conditions, including hypertension, diabetes, coronary heart disease, congestive heart failure, and stroke; 5). Medications, including diuretics, angiotensin-converting enzyme inhibitors, angiotensin II inhibitors, and systemic corticosteroids; and 6).Kidney measures, including estimated glomerular filtration rate calculated by the cystatinC-based Chronic Kidney Disease Epidemiology Collaboration Equations^58^ and urine albumin-to-creatinine ratio which was categorized into normal, microalbuminuria, and macroalbuminuria. Details of these measurements can be found in the UK BiobankData Showcase (http://biobank.ctsu.ox.ac.uk/crystal/).

### Quantification and statistical analysis

Baseline characteristics are presented in the study population as a whole and according to quartiles of estimated 24-h urinary sodium excretion. For ESKD, the median (IQR) follow-up period, the number of events occurred, and the annual incidence rates were calculated.

We used multiple imputation with chained equations to address missing data. The number of multiple imputations was five. The imputation method for continuous data was predictive mean matching (PMM); for binary data was logistic regression (Log-Reg); for unordered categorical data (factor >2 levels) was polytomous regression (PolyReg); and for ordinal data was proportional odds model (Polr). We pooled the estimates from Cox proportional hazards models across imputed datasets using Rubin’s rules.^59^

The proportional hazards assumption was evaluated using Schoenfeld residuals, and if it is not fulfilled the follow-up time was split accordingly. Multicollinearity was assessed in each model with the variance inflation factor. The variables with variance inflation factor values above 5 were considered to have severe collinearity and were excluded except for one variable that was the most relevant to the outcome in a biological sense.

In addition, we used the McMahon-Peto method to address the regression dilution bias.^15^ We calculated estimated 24-h urinary sodium excretion based on the repeated measurements of spot urinary biomarker concentrations. Participants were assigned to quintiles according to the rank of estimated 24-h urinary sodium excretion in the first measurement. The regression dilution ratio was obtained by dividing the difference in the mean estimated 24-h urinary sodium excretion between the 5^th^ and the 1^st^ quintiles in the second measurement by the equivalent mean difference in the first measurement. Then, the log(HR)s and standard errors were adjusted by dividing the regression dilution ratios.

We constructed three sequential models. Model 0 was a crude model. Model 1 was adjusted for baseline demographic (age, sex, ethnicity, education, and Townsend deprivation index) and lifestyle factors (smoking, alcohol consumption, physical activity, and estimated 24-h urinary potassium excretion). Model 2 was additionally adjusted for anthropometric measurements (waist circumference), comorbid conditions (hypertension, diabetes, coronary heart disease [CHD], congestive heart failure [CHF], and stroke), medications (diuretics and angiotensin-converting enzyme inhibitors [ACEIs]/angiotensin II receptor blockers [ARBs]), and baseline estimated glomerular filtration rate (eGFR).

We did not adjust for baseline systolic and diastolic blood pressure (SBP/DBP), and urine albumin-to-creatinine ratio (UACR). According to previous literature,^1^^,^^10^ sodium restriction has been shown to have effects on lowering blood pressure and urinary protein excretion (as stated in Introduction). Therefore, SBP, DBP, and UACR were considered as potential mediators rather than confounders, thus were not adjusted.

We treated hypertension and anti-hypertensive medications as confounders because they represented the condition prior to the baseline. For this reason, the temporal order suggested that they were more likely to be confounders. A history of hypertension and anti-hypertensive medications may affect the urinary sodium excretion estimated at baseline due to the following reasons: 1) some people with existing hypertension may follow medical advice to reduce sodium intake; 2) certain anti-hypertensive medications (eg, diuretics, ACEIs/ARBs) could affect urinary sodium excretion under the pharmacological mechanism. On the other hand, we acknowledge the possibility that some people might have consumed a high amount of sodium prior to developing hypertension/taking anti-hypertensive medications and continued to do so even after hypertension diagnosis until the recruitment. In this scenario, hypertension history and anti-hypertensive medications might play a mediating role (Figure S2). To rule out the possibility of over-adjustment in Model 2, we added a sensitivity analysis, in which we removed the history of hypertension and anti-hypertensive medications from the covariates of Model 2.

Body mass index was removed from the final Model 2 fitted because of multicollinearity. Estimated 24-h urinary sodium excretion was entered into the models as 1) a continuous variable without transformation; 2) a binary variable (<2 g vs. ≥ 2 g, where sodium intake of less than 2 g/d is recommended by the World Health Organization)^1^; and 3) a discrete variable constructed based on quartiles. Additionally, the potential nonlinear relationship between estimated 24-h urinary sodium excretion and incident ESKD was evaluated on a continuous scale with restricted cubic spline curves based on Cox proportional hazards models.

Furthermore, to assess the robustness of the results, we performed several sensitivity analyses as well as a sex-specific subgroup analysis. First, since certain medications might interfere with the results of urinary biomarkers, we excluded participants who took diuretics, ACEIs, ARBs, or glucocorticoids at baseline. Second, we conducted analyses using estimated 24-h urinary sodium excretion calculated by the Kawasaki equations as the exposure. Third, to tackle potential pitfalls of estimated 24-h urinary sodium excretion using formulas, we treated urinary sodium-to-potassium ratio as a substitute for the exposure. Fourth, to reduce the risk of reverse causation, we excluded participants who had hypertension, CHD, CHF, stroke, CKD stage 3-5 (eGFR<60 ml/min/1.73m^2^) or albuminuria (UACR ≥30 mg/g) at baseline, and who developed ESKD or died within the initial 2 years of follow-up. Fifth, in order to take competing risks - deaths prior toESKD - into account, we used the Fine and Gray approach (subdistribution hazards models) to access the association studied. Sixth, we conducted the analysis with the original data, i.e., without multiple imputation. Seventh, to rule out the possibility of over-adjustment in Model 2, we removed the history of hypertension and anti-hypertensive medications from the covariates. Eighth, we additionally adjusted for processed meat intake and phosphate on the basis of Model 2. Last, to test potential sex difference, we repeated the analysis stratified by sex.

All statistical analyses were performed using R Statistical Software, version 4.1.3. Multiple imputation was implemented using the R package mice. All statistical tests were two-sided, and we considered a P value less than 0.05 to be statistically significant.

## Data Availability

This paper analyzes existing, publicly available data. These accession numbers for the datasets are listed in the key resources table. All original code has been deposited at Zenodo and is publicly available as of the date of publication. DOIs are listed in the key resources table. Any additional information required to reanalyze the data reported in this paper is available from the lead contact upon request.
