# Supplementary material for: Estimated 24-h urinary sodium excretion and risk of end-stage kidney disease
Source: iScience. 2023 Apr 23;26(5):106728. doi: 10.1016/j.isci.2023.106728 (PMC10192648; doi:10.1016/j.isci.2023.106728)
Supplement: Document S1. Figures S1 and S2 and Tables S1–S10 [file mmc1.pdf]

iScience, Volume *26*

Supplemental information

Estimated 24-h urinary sodium excretion

and risk of end-stage kidney disease

Ying Shan, Yong Bai, Jingwen Zhang, Yueqi Lu, Sike Yu, Congying Song, Juehan Liu, Min Jian, Junjie Xu, Changhai Ding, Zuying Xiong, and Xiaoyan Huang

SUPPLEMENTARY

Figure S1. Histograms of the estimated 24-h urinary sodium excretion, related to Results......2
Figure S2. A directed acyclic graph demonstrating the relationship of hypertension history, anti-hypertensive medication usage, systolic and diastolic blood pressure (SBP/DBP), urine albumin-to-creatinine ratio (UACR), sodium intake/urinary sodium excretion and end stage kidney disease (ESKD), related to STAR Methods. 3
Table S1. Adjusted hazard ratios for end-stage kidney disease under sensitivity analysis 1, related to Results..4
Table S2. Adjusted hazard ratios for end-stage kidney disease under sensitivity analysis 2, related to Results..5
Table S3. Adjusted hazard ratios for end-stage kidney disease under sensitivity analysis 3, related to Results..6
Table S4. Adjusted hazard ratios for end-stage kidney disease under sensitivity analysis 4, related to Results..7
Table S5. Adjusted hazard ratios for end-stage kidney disease under sensitivity analysis 5, related to Results..8
Table S6. Adjusted hazard ratios for end-stage kidney disease under sensitivity analysis 6, related to Results..9
Table S7. Adjusted hazard ratios for end-stage kidney disease under sensitivity analysis 7, related to Results..10
Table S8. Adjusted hazard ratios for end-stage kidney disease under sensitivity analysis 8, related to Results..11
Table S9. Adjusted hazard ratios for end-stage kidney disease stratified by sex, related to Results..12
Table S10. Data-field IDs used in this study and the corresponding descriptions in the UK Biobank, related to STAR Methods. 13

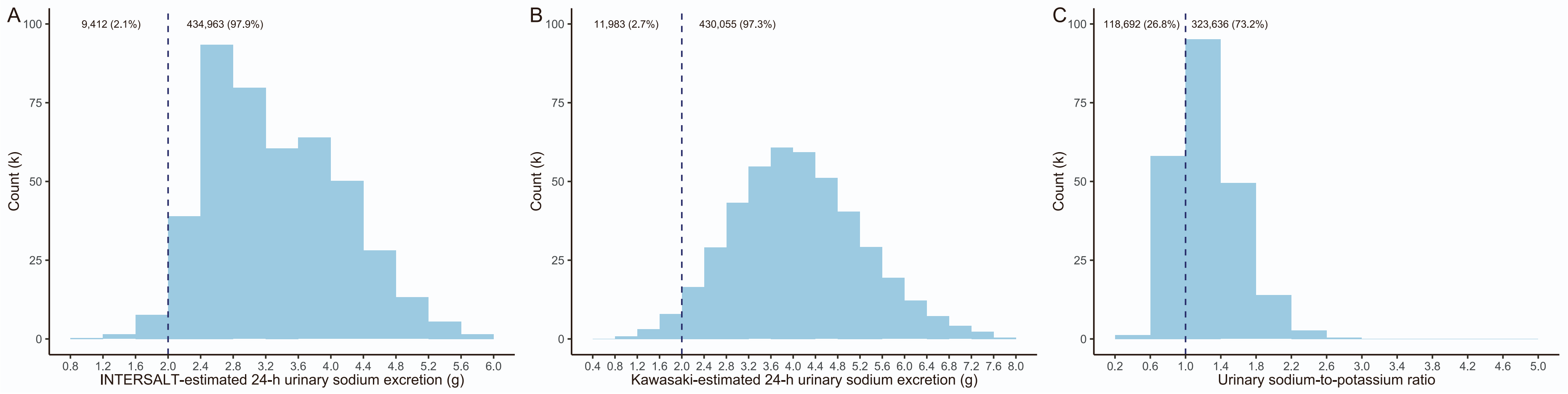

Figure S1. Histograms of the estimated 24-h urinary sodium excretion, related to Results.

Panel a and b, estimated 24-h urinary sodium excretion calculated by the INTERSALT (a) and Kawasaki (b) equations, respectively. The vertical dashed lines indicate the upper limit of daily sodium intake (2 g) recommended by the World Health Organization. Panel c, the ratio of estimated 24-h urinary sodium and potassium excretion. The vertical dashed line indicates the ratio of 1.

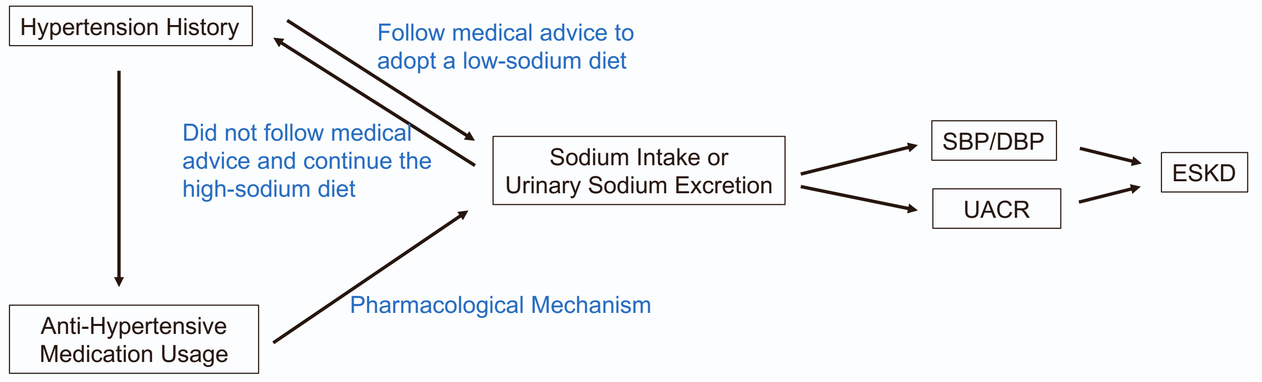

Figure S2. A directed acyclic graph demonstrating the relationship of hypertension history, anti-hypertensive medication usage, systolic and diastolic blood pressure (SBP/DBP), urine albumin-to-creatinine ratio (UACR), sodium intake/urinary sodium excretion and end-stage kidney disease (ESKD), related to STAR Methods.

Table S1. Adjusted hazard ratios for end-stage kidney disease under sensitivity analysis 1, related to Results.

Estimated 24-h urinary sodium excretion	HRs (95% CIs)	RDR-adjusted HRs (95% CIs)	P
Continuous, per 1 g increment	0.95 (0.76, 1.18)	0.94 (0.72, 1.22)	0.650
Binary			
Below 2 g	Reference	Reference	
Above 2 g	1.36 (0.63, 2.93)	1.45 (0.58, 3.63)	0.433
Multicategory			
Quartile 1	Reference	Reference	
Quartile 2	1.13 (0.78, 1.64)	1.16 (0.74, 1.81)	0.511
Quartile 3	1.22 (0.79, 1.89)	1.27 (0.76, 2.15)	0.363
Quartile 4	1.12 (0.67, 1.86)	1.14 (0.62, 2.10)	0.665
P for linear trend	0.625		
P for quadratic trend	0.372		

Participants who took diuretics, angiotensin-converting enzyme inhibitors, angiotensin II receptor blockers, or glucocorticoids were excluded, leaving 360,776 individuals for this sensitivity analysis.

Models were adjusted for age, sex, ethnicity, Townsend deprivation index, education, smoking status, alcohol consumption, physical activity, estimated 24-h urinary potassium excretion, waist circumference, hypertension history, diabetes history, coronary heart disease history, congestive heart failure history, stroke history, and estimated glomerular filtration rate. The regression dilution ratio was 0.83.

Abbreviations: CIs, confidence intervals; RDR, regression dilution ratio; HRs, hazard ratios.

Table S2. Adjusted hazard ratios for end-stage kidney disease under sensitivity analysis 2, related to Results.

Estimated 24-h urinary sodium excretion	HRs (95% CIs)	RDR-adjusted HRs (95% CIs)	P
Continuous, per 1 g increment	1.03 (0.97, 1.09)	1.11 (0.91, 1.36)	0.293
Binary			
Below 2 g	Reference	Reference	
Above 2 g	1.28 (0.93, 1.75)	2.30 (0.80, 6.65)	0.124
Multicategory			
Quartile 1	Reference	Reference	
Quartile 2	0.94 (0.78, 1.14)	0.82 (0.43, 1.58)	0.557
Quartile 3	1.06 (0.87, 1.29)	1.21 (0.63, 2.35)	0.564
Quartile 4	0.99 (0.81, 1.21)	0.96 (0.48, 1.91)	0.913
P for linear trend	0.716		
P for quadratic trend	0.896		

Sex-specific Kawasaki equations were employed to estimate 24-h urinary sodium excretion for this sensitivity analysis.

Models were adjusted for age, sex, ethnicity, Townsend deprivation index, education, smoking status, alcohol consumption, physical activity, estimated 24-h urinary potassium excretion, waist circumference, hypertension history, diabetes history, coronary heart disease history, congestive heart failure history, stroke history, diuretics, angiotensin-converting enzyme inhibitors or angiotensin II receptor blockers, and estimated glomerular filtration rate. The regression dilution ratio was 0.30.

Abbreviations: CIs, confidence intervals; RDR, regression dilution ratio; HRs, hazard ratios.

Table S3. Adjusted hazard ratios for end-stage kidney disease under sensitivity analysis 3, related to Results.

The ratio of urinary sodium to potassium	HRs (95% CIs)	RDR-adjusted HRs (95% CIs)	P
Continuous, per 1 increment	1.13 (0.86, 1.48)	1.28 (0.74, 2.24)	0.377
Binary			
Below 1	Reference	Reference	
Above 1	1.00 (0.79, 1.27)	1.00 (0.62, 1.62)	0.999
Multicategory			
Quartile 1	Reference	Reference	
Quartile 2	1.02 (0.78, 1.33)	1.04 (0.61, 1.78)	0.884
Quartile 3	1.03 (0.78, 1.36)	1.06 (0.61, 1.87)	0.828
Quartile 4	1.21 (0.88, 1.65)	1.47 (0.78, 2.78)	0.234
P for linear trend	0.231		
P for quadratic trend	0.347		

The ratio of urinary sodium and potassium excretion was used as the exposure of interest for this sensitivity analysis.

Models were adjusted for age, sex, ethnicity, Townsend deprivation index, education, smoking status, alcohol consumption, physical activity, waist circumference, hypertension history, diabetes history, coronary heart disease history, congestive heart failure history, stroke history, diuretics, angiotensin-converting enzyme inhibitors or angiotensin II receptor blockers, and estimated glomerular filtration rate. The regression dilution ratio was 0.49.

Abbreviations: CIs, confidence intervals; RDR, regression dilution ratio; HRs, hazard ratios.

Table S4. Adjusted hazard ratios for end-stage kidney disease under sensitivity analysis 4, related to Results.

Estimated 24-h urinary sodium excretion	HRs (95% CIs)	RDR-adjusted HRs (95% CIs)	P
Continuous, per 1 g increment	0.86 (0.61, 1.21)	0.83 (0.55, 1.26)	0.388
Binary			
Below 2 g	Reference	Reference	
Above 2 g	1.48 (0.36, 6.09)	1.60 (0.29, 8.81)	0.589
Multicategory			
Quartile 1	Reference	Reference	
Quartile 2	1.21 (0.70, 2.11)	1.26 (0.65, 2.46)	0.492
Quartile 3	1.08 (0.55, 2.13)	1.10 (0.49, 2.49)	0.814
Quartile 4	1.01 (0.46, 2.19)	1.01 (0.40, 2.57)	0.982
P for linear trend	0.949		
P for quadratic trend	0.455		

Participants with prevalent hypertension (systolic blood pressure ≥ 140 mmHg or diastolic blood pressure ≥ 90 mmHg or use of antihypertensive medication), congestive heart failure, coronary heart disease, stroke, chronic kidney disease stage 3-5 (estimated glomerular filtration rate < 60 ml/min/1.73m^2), or albuminuria (urine albumin-creatinine ratio ≥ 30mg/g) at baseline, as well as who developed end-stage kidney disease or died within the initial 2 years of follow-up, were excluded, leaving 297,893 individuals for this sensitivity analysis.

Models were adjusted for age, sex, ethnicity, Townsend deprivation index, education, smoking status, alcohol consumption, physical activity, estimated 24-h urinary potassium excretion, waist circumference, diabetes, diuretics, angiotensin-converting enzyme inhibitors or angiotensin II receptor blockers, and estimated glomerular filtration rate. The regression dilution ratio was 0.83.

Abbreviations: CIs, confidence intervals; RDR, regression dilution ratio; HRs, hazard ratios.

Table S5. Adjusted hazard ratios for end-stage kidney disease under sensitivity analysis 5, related to Results.

Estimated 24-h urinary sodium excretion	HRs (95% CIs)	RDR-adjusted HRs (95% CIs)	P
Continuous, per 1 g increment	1.06 (0.95, 1.19)	1.07 (0.94, 1.23)	0.264
Binary			
Below 2 g	Reference	Reference	
Above 2 g	1.54 (0.95, 2.50)	1.67 (0.94, 2.97)	0.080
Multicategory			
Quartile 1	Reference	Reference	
Quartile 2	1.01 (0.79, 1.28)	1.01 (0.76, 1.34)	0.949
Quartile 3	1.15 (0.91, 1.46)	1.18 (0.89, 1.57)	0.250
Quartile 4	1.19 (0.91, 1.56)	1.23 (0.89, 1.70)	0.214
P for linear trend	0.155		
P for quadratic trend	0.879		

The Fine and Gray approach was applied for this sensitivity analysis, in order to consider deaths prior to end-stage kidney disease as the competing risks.

Models were adjusted for age, sex, ethnicity, Townsend deprivation index, education, smoking status, alcohol consumption, physical activity, estimated 24-h urinary potassium excretion, waist circumference, hypertension history, diabetes history, coronary heart disease history, congestive heart failure history, stroke history, diuretics, angiotensin-converting enzyme inhibitors or angiotensin II receptor blockers, and estimated glomerular filtration rate. The regression dilution ratio was 0.84.

Abbreviations: CIs, confidence intervals; RDR, regression dilution ratio; HRs, hazard ratios.

Table S6. Adjusted hazard ratios for end-stage kidney disease under sensitivity analysis 6, related to Results.

Estimated 24-h urinary sodium excretion	HRs (95% CIs)	RDR-adjusted HRs (95% CIs)	P
Continuous, per 1 g increment	1.08 (0.94, 1.25)	1.10 (0.92, 1.31)	0.285
Binary			
Below 2 g	Reference	Reference	
Above 2 g	1.11 (0.64, 1.91)	1.13 (0.59, 2.16)	0.713
Multicategory			
Quartile 1	Reference	Reference	
Quartile 2	0.98 (0.73, 1.30)	0.97 (0.69, 1.37)	0.884
Quartile 3	1.06 (0.77, 1.45)	1.07 (0.74, 1.55)	0.724
Quartile 4	1.17 (0.82, 1.66)	1.20 (0.79, 1.83)	0.391
P for linear trend	0.366		
P for quadratic trend	0.497		

We used the original data without multiple imputation, leaving 333,711 individuals for this sensitivity analysis.

Models were adjusted for age, sex, ethnicity, Townsend deprivation index, education, smoking status, alcohol consumption, physical activity, estimated 24-h urinary potassium excretion, waist circumference, hypertension history, diabetes history, coronary heart disease history, congestive heart failure history, stroke history, diuretics, angiotensin-converting enzyme inhibitors or angiotensin II receptor blockers, and estimated glomerular filtration rate. The regression dilution ratio was 0.84.

Abbreviations: CIs, confidence intervals; RDR, regression dilution ratio; HRs, hazard ratios.

Table S7. Adjusted hazard ratios for end-stage kidney disease under sensitivity analysis 7, related to Results.

Estimated 24-h urinary sodium excretion	HRs (95% CIs)	RDR-adjusted HRs (95% CIs)	P
Continuous, per 1 g increment	1.08 (0.96, 1.22)	1.09 (0.95, 1.27)	0.201
Binary			
Below 2 g	Reference	Reference	
Above 2 g	1.48 (0.91, 2.42)	1.59 (0.89, 2.86)	0.118
Multicategory			
Quartile 1	Reference	Reference	
Quartile 2	0.99 (0.78, 1.26)	0.99 (0.75, 1.31)	0.951
Quartile 3	1.12 (0.87, 1.45)	1.14 (0.85, 1.55)	0.375
Quartile 4	1.20 (0.89, 1.60)	1.23 (0.87, 1.75)	0.231
P for linear trend	0.184		
P for quadratic trend	0.633		

In this analysis, we removed the history of hypertension, as well as the anti-hypertensive medications from the covariates.

Models were adjusted for age, sex, ethnicity, Townsend deprivation index, education, smoking status, alcohol consumption, physical activity, estimated 24-h urinary potassium excretion, waist circumference, diabetes history, coronary heart disease history, congestive heart failure history, stroke history, and estimated glomerular filtration rate. The regression dilution ratio was 0.84.

Abbreviations: CIs, confidence intervals; RDR, regression dilution ratio; HRs, hazard ratios.

Table S8. Adjusted hazard ratios for end-stage kidney disease under sensitivity analysis 8, related to Results.

Estimated 24-h urinary sodium excretion	HRs (95% CIs)	RDR-adjusted HRs (95% CIs)	P
Continuous, per 1 g increment	1.11 (0.97, 1.27)	1.13 (0.97, 1.33)	0.272
Binary			
Below 2 g	Reference	Reference	
Above 2 g	1.42 (0.84, 2.40)	1.52 (0.81, 2.84)	0.189
Multicategory			
Quartile 1	Reference	Reference	
Quartile 2	0.94 (0.72, 1.23)	0.93 (0.68, 1.27)	0.647
Quartile 3	1.11 (0.83, 1.47)	1.13 (0.80, 1.58)	0.489
Quartile 4	1.18 (0.85, 1.63)	1.22 (0.83, 1.78)	0.318
P for linear trend	0.233		
P for quadratic trend	0.442		

In this analysis, we additionally adjusted for processed meat intake and phosphate on the basis of Model 2.

Models were adjusted for age, sex, ethnicity, Townsend deprivation index, education, smoking status, alcohol consumption, physical activity, estimated 24-h urinary potassium excretion, waist circumference, hypertension history, diabetes history, coronary heart disease history, congestive heart failure history, stroke history, diuretics, angiotensin-converting enzyme inhibitors or angiotensin II receptor blockers, estimated glomerular filtration rate, processed meat intake and phosphate. The regression dilution ratio was 0.84.

Abbreviations: CIs, confidence intervals; RDR, regression dilution ratio; HRs, hazard ratios.

Table S9. Adjusted hazard ratios for end-stage kidney disease stratified by sex, related to Results.

Estimated 24-h urinary sodium excretion, per 1 g increment	n (event)	HRs (95% CIs)	RDR-adjusted HRs (95% CIs)	P for difference
Women	240,241 (306)	0.85 (0.65,1.11)	0.77 (0.50,1.19)	0.07
Men	204,134 (559)	1.07 (0.93,1.23)	1.14 (0.86,1.52)	

Models were adjusted for age, ethnicity, Townsend deprivation index, education, smoking status, alcohol consumption, physical activity, estimated 24-h urinary potassium excretion, waist circumference, hypertension history, diabetes history, coronary heart disease history, congestive heart failure history, stroke history, diuretics, angiotensin-converting enzyme inhibitors or angiotensin II receptor blockers, and estimated glomerular filtration rate. The regression dilution ratios were 0.49 and 0.63 for women and men, respectively.

P for difference between sex-specific associations was assessed using a 2-sample z-test.

Abbreviations: CIs, confidence intervals; RDR, regression dilution ratio; HRs, hazard ratios.

Table S10. Data-field IDs used in this study and the corresponding descriptions in the UK Biobank, related to STAR Methods.

Data-Field	Description
31	Sex
48	Waist circumference
53	Date of attending assessment centre
189	Townsend deprivation index at recruitment
191	Date lost to follow-up
1349	Processed meat intake
2443	Diabetes diagnosed by doctor
6138	Education
6150	Vascular/heart problems diagnosed by doctor
12144	Height
20003	Treatment/medication code
20116	Smoking status
20117	Alcohol drinker status
21000	Ethnic background
21001	Body mass index (BMI)
21022	Age at recruitment
22032	IPAQ activity group
30500	Microalbumin in urine
30505	Microalbumin in urine result flag
30510	Creatinine (enzymatic) in urine
30520	Potassium in urine
30530	Sodium in urine
30700	Creatinine
30720	Cystatin C
30810	Phosphate
40000	Date of death
40005	Date of cancer diagnosis
40012	Behaviour of cancer tumour
41270	Diagnoses - ICD10
41271	Diagnoses - ICD9
41280	Date of first in-patient diagnosis - ICD10
42026	Date of end stage renal disease report
